# Alternative splicing and MicroRNA: epigenetic mystique in male reproduction

**DOI:** 10.1080/15476286.2021.2024033

**Published:** 2022-01-22

**Authors:** Di Wu, Faheem Ahmed Khan, Lijun Huo, Fei Sun, Chunjie Huang

**Affiliations:** a Institute of Reproductive Medicine, School of Medicine, Nantong University, Nantong, China; b Laboratory of Molecular Biology and Genomics, Department of Zoology, Faculty of Science, University of Central Punjab, Lahore, Pakistan; c College of Animal Science and Technology, Huazhong Agricultural University, Wuhan, China

**Keywords:** Male fertility, spermatogenesis, alternative splicing, miRNA, epigenetic inheritance

## Abstract

Infertility is rarely life threatening, however, it poses a serious global health issue posing far-reaching socio-economic impacts affecting 12–15% of couples worldwide where male factor accounts for 70%. Functional spermatogenesis which is the result of several concerted coordinated events to produce sperms is at the core of male fertility, Alternative splicing and microRNA (miRNA) mediated RNA silencing (RNAi) constitute two conserved post-transcriptional gene (re)programming machinery across species. The former by diversifying transcriptome signature and the latter by repressing target mRNA activity orchestrate a spectrum of testicular events, and their dysfunctions has several implications in male infertility. This review recapitulates the knowledge of these mechanistic events in regulation of spermatogenesis and testicular homoeostasis. In addition, miRNA payload in sperm, vulnerable to paternal inputs, including unhealthy diet, infection and trauma, creates epigenetic memory to initiate intergenerational phenotype. Naive zygote injection of sperm miRNAs from stressed father recapitulates phenotypes of offspring of stressed father. The epigenetic inheritance of paternal pathologies through miRNA could be a tantalizing avenue to better appreciate ‘Paternal Origins of Health and Disease’ and the power of tiny sperm.

## Introduction

**Figure 1.**
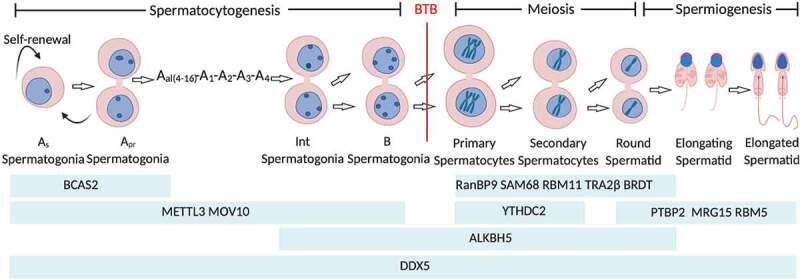
Schematic representation of mouse spermatogenesis, and the expression of key alternative splicing factors. Asingle (As; isolated single cells] spermatogonia can either proceed mitotically self-renew (stemness maintenance) or differentiate into Apaired (Apr; chains of two cells) and Aaligned (Aal; chains of 4, 8, 16 or 32 cells) spermatogonia which are collectively referred to as undifferentiated spermatogonia. Apr spermatogonia however renders the potential of reprogramming into ‘true’ stem cell via dedifferentiation [164]. While Aal spermatogonia, with the intervening of A1-A4 intermediates, then develops into the precursor B spermatogonia. The type B spermatogonia further differentiates into preleptotene spermatocyte which commences two constitutive meiotic divisions to yield haploid spherical spermatid. Spermiogenesis or haploid spermatid differentiation is the final stage of spermatogenesis. Spermatid undergoes a series of morphological changes, such as elongation, nuclear remodelling, acrosome formation and flagellum. BTB: blood–testis barrier.

Until puberty, the exquisite stem cell driven spermatogenesis in mammal is initiated to continuously produce haploid male gametes [1]. Spermatogenesis involves three sequential processes: (i) spermatocytogenesis: whereby spermatogonia stem cells undergo mitotical self-renewal and differentiation into primary spermatocytes; (ii) nuclear maturation: wherein spermatocytes commence two constitutive meiotic divisions without an interval of DNA synthesis to yield haploid spherical spermatids; (iii) spermiogenesis: a process of stepwise morphogenetic changes of spherical spermatids prior to being released into seminiferous tubule lumen as spermatozoa (Fig. 1). Further epididymal maturation renders spermatozoa the fertilization competency [2]. Dysfunction in any process will impair male fertility with compromised semen quality, such as azoospermia, oligozoospermia, necrozoospermia and teratozoospermia [11^3^].

**Figure 2.**
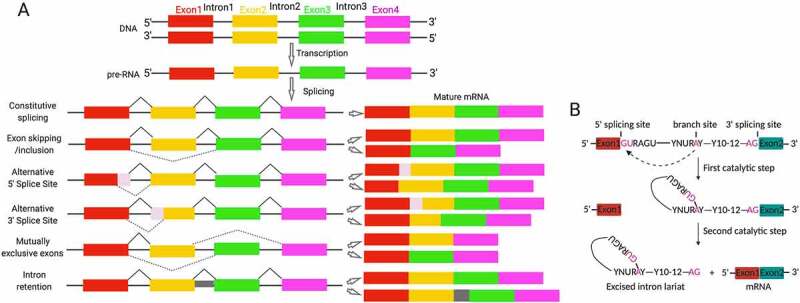
Mechanisms of alternative splicing. (A) Illumination of alternative splicing patterns that produce transcript variants. Coloured boxes indicate exons while lines represent intron. The dotted lines indicate alternative splicing processes of exon skipping/inclusion, alternative 5ʹ splice sites, alternative 3ʹ splice sites, mutually exclusive exons and intron retention. Retained introns occur with the absence of splicing with intervening intron (grey) is included in final transcript. (B) The chemical process of splicing. Splicing sites within the intron are shown. Specifically, the adenosine in branch site attracts the phosphodiester bond within 5ʹ splicing site during the first transesterification. In the second step, 5ʹ exon attacks the phosphodiester bond within 3ʹ splicing site followed by exon fusion and the lariat-structured intron is released as lariat product.

The crunch of biology is that DNA decodes phenotype. However, the aetiology of diseases including infertility in most cases is non-DNA sequence-associated [1, 4]. Beyond alternation in DNA sequence, genomic DNA function can be fine tuned from transcription to post-translation by epigenetic mechanisms, including RNA editing by alternative splicing and non-coding RNAs. Across species, proteins overwhelmingly outnumber protein-encoding genes in a genome. Alternative pre-mRNA splicing constitutes the most important source of proteome diversity contributing to the identity, development, diversity and specificity of the cell [5–7], and its dysfunction is associated with multiple human diseases [8,9]. Splicing machinery functions as a proteinous ribonucleoprotein (RNP) complex, namely spliceosome [10]. The spliceosome is a ribozyme assembled of five small nuclear ribonucleoproteins (snRNP) particles and numerous proteins that removes introns in pre-mRNA to generate functionally distinct mRNA variants [11]. The patterns and chemical processes of alternative splicing are illustrated in Fig. 2. Strikingly, the testis ranks among top tissues in regard to the complexity of mRNA variants [12]. A wealth of splicing factors that regulate mRNA stability, localization and signalling-dependent translation have emerged as determinants for spermatogenesis and male fertility (Fig. 1).

**Figure 3.**
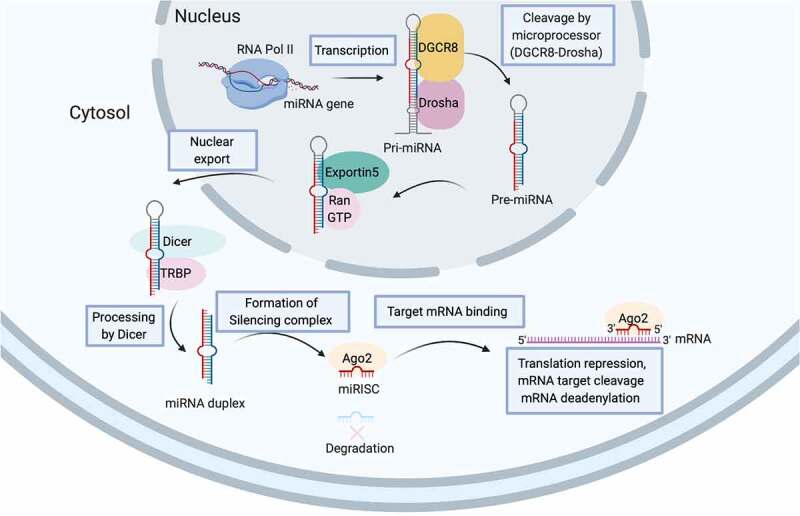
miRNA biogenesis. The biogenesis of miRNAs begins with the transcription of a double-stranded, hairpin-formed primary miRNA transcript (pri-miRNA) by RNA polymerase II (RNA Pol II). The microprocessor complex composing of RNA-binding protein DGCR8 and RNase-III enzyme Drosha catalyzes the hairpin loop and liberates the ~85-nucleotide stem-loop structured miRNA precursors (pre-miRNA) which is then exported from nucleus by Exportin 5-Ran-GTP complex to cytoplasm. RNase-III enzyme Dicer further cleaves off the terminal loop and generates the 20–22 nucleotide double-stranded intermediate. The functional strand of mature miRNA (red) together with numerous proteins including those of Argonaute (Ago) family are assemble to form the functional miRNA-induced silencing complex (miRISC) to silence the expression of target gene (blue) with complementary sequences.

Unlike alternative splicing that diversifies mRNA variants, non-coding RNAs (ncRNAs), the abundant functional RNA transcripts without protein-coding property, are tire post-transcriptional regulators of gene expression. The landscape of ncRNAs has not yet fully deciphered but novel subspecies is emerging [13]. miRNA together with small interfering RNAs (siRNA) and PIWI-associated RNAs (piRNAs) are subspecies of small non-coding RNAs (sncRNAs) involving in the genetic regulatory process termed RNA silencing [14]. The biogenesis of miRNA is illustrated in Fig. 3. miRNA matures from its pre-miRNA precursor and afterwards is assembled into the functional RNP complex which is referred to as miRNA-induced silencing complexes (miRISCs) for fine-tuning expression of target gene with complementary sequence (Fig. 3). Beyond canonically regulates cytosolic mRNA stability or translation, miRNA likely regulates pre-mRNA editing or functions as chaperone that influences mRNA structure or mRNA-protein interaction [14]. In addition, miRNA constitutes a key make-up of extracellular vesicle for intercellular or inter-organ crosstalk [14,15], which replenishes our understanding of health and disease. Apart from its reckoned importance in spermatogenesis (Table 1), miRNA in paternal gamete is preserved as important epigenetic code for embryonic development as embryo derived from sperm depleted for *Dicer*, a necessary endoribonuclease for miRNA biogenesis, has preimplantation development defects that can be rescued by injection of naive sperm RNAs [16].

**Table 1.** The importance of miRNA in testicle functionality

miRNA	Species/cell origin	Target/associated genes	Function	Reference
miR-10a	Mouse male germ cell	Rad51	DSB repair	150
miR-125b-2	Mouse male germ cell	Pap	Testosterone secretion	151
miR-663a	Human spermatogonial stem cell	Nfix	Cell cycle and DNA synthesis	117
miR-202	Mouse spermatogonial stem cell	Rbfox2	Spermatogonial stem cell maintenance	152
miR-224	Mouse spermatogonial stem cell	Dmrt1	Spermatogonial stem cell self-renewal	153
miR-34b/c/449	Post-mitotic male germ cell	E2F-pRb pathway	Meiosis and spermatozoa maturation	154 155
miR-135a	Rat spermatogonial stem cell	Foxo1	Spermatogonial stem cell maintenance	156
miR-221/222	Mouse Thy^+^ spermatogonial stem cell	Kit	Undifferentiated spermatogonia maintenance	[157]
miR-469	Pachytene spermatocyte/spherical spermatid	Tp2 and Prm2	Chromatin remodelling	158
miR-21	Mouse Thy^+^ spermatogonial stem cell	unknown	Undifferentiated spermatogonia maintenance	159
Fragile-X-miR cluster	Sertoli cell	Fmr1	Translation of batteries of mRNAs	160
miR-202-3p	Sertoli cell	Lrp6 and Cyclin D1	Proliferation, synthesis function and apoptosis	[161]
miR-320-3p	Sertoli cell	Glut3	Lactate metabolism	162
miR-142-3p	Sertoli cell	Lgl2	BTB integrity	163
miR-471-5p	Sertoli cell	Dock180, Lc3, Atg12, Becn1	Phagocytosis of apoptotic germ cells	142

The concept of ‘Developmental Origins of Health and Disease’ captures the influences on health in adulthood of foetal life experience [17]. Intensive studies have demonstrated that maternal inputs during or prior to pregnancy, such as obesity, predispose offspring to disease susceptibility and sometimes over generations [18]. Recent evidence suggests paternal inputs, such as unhealthy diet, infection and trauma, also have intergenerational consequences by reprogramming sperm epigenome [19–21]. Deciphering epigenetic codes for specific transmitted phenotype is challenging partially due to complexity in epigenetic reprogramming, namely DNA methylation, histone modifications, small non-coding RNAs (sncRNAs) and its modifications [4]. Mammalian sperm is abundant in sncRNAs, including miRNA and unique tRNA-derived small RNA (tsRNA) [13]. In addition, sperm receives sncRNAs from reproductive tracts derived extracellular vesicles [15,22]. Of primary interest is miRNA, which is highly sensitive to environmental stimuli and underpins transmission of paternal pathologies, and zygote injection of sperm miRNAs of stressed male recapitulates pathologies of offspring of stressed male [20,21]. These phenomena conceptualize ‘Paternal Origin of Health and Disease’ and captivate investigations of the underlying epigenetic codes [17]. Given the prevalence of infertility and metabolic diseases, a detailed understanding of the epigenetic landscape in generation of a ‘healthy’ sperm will revolutionize clinical innovations for fertility improvement, preconception diagnosis and the birth of a ‘healthier’ baby.

## Alternative splicing in spermatogonia stem cells (SSCs) homoeostasis

Spermatogonial stem cells (SSCs), the heterogeneous undifferentiated spermatogonia comprising 0.01–1% of testicle cell populations, host properties of stemness maintenance and differentiation into spermatocytes that commence meiosis. Spermatogonia differentiation is under fine-tuning regulation of a myriad of signalling pathways [23]. In particular, spermatogonia displays exceptional transcriptome diversity [24], linking alternative splicing with SSCs homoeostasis, especially during SSCs ageing. The attenuated differentiation of aged SSCs is accompanied by alteration in alternative splicing machinery and differential expression of specific lncRNAs [25]. Breast carcinoma amplified sequence2 (BCAS2) a splicing factor originally characterized in human breast cancer cells has specifically enrichment in spermatogonia. While spermatogonia undergoes normal proliferation and apoptosis in *Bcas2* null mice, its further differentiation is dampened [26]. Epithelial splicing regulatory protein 1 (ESRP1) also highly enriches in type A and B spermatogonia in a speckled pattern [27]. The involvement of ESRP1 in alternative splicing has been studied in spermatogonia cell line, but *in vivo* study still needs to better characterize its relevance.

**Figure 4.**
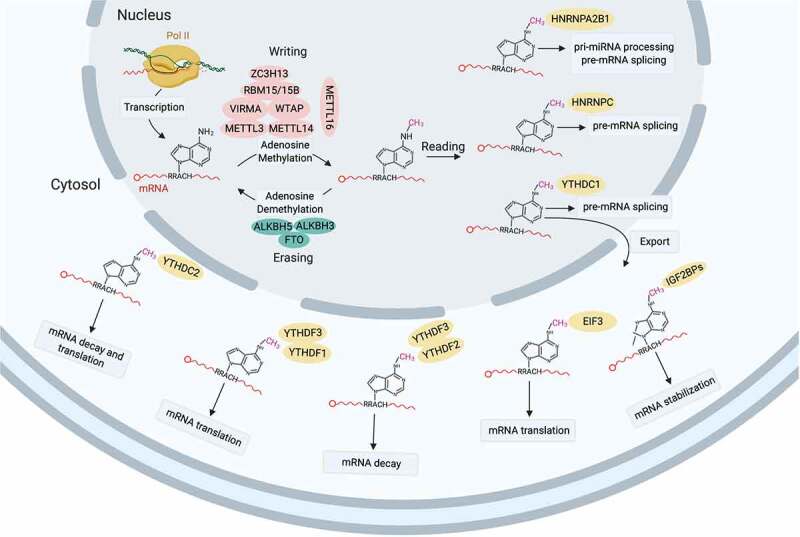
A summarized landscape of m^6^A in RNA biology. N6-methyladenosine (m^6^A) modification is catalysed by the ‘writer’ (methyltransferase-like protein (METTL)3-METTL14 heterodimer) in the nucleus with the complex comprising WTAP, VIRMA, ZC3H13 and RBM15/15B functions as a regulatory element. METTL16 is a novel independent RNA methyltransferase that catalyzes m^6^A modification of U6-snRNA a component of alternative splicing machinery. m^6^A modification is removed by the ‘eraser’ (demethtlases FTO, ALKBH5 and ALKBH3) and recognized by various m6A-specific ‘reader’ (YTHDC1/2, YTHDF1/2/3, IGF2BP1/2/3, HNRNPs and eIF3), which determine the fate of target mRNA: alternative splicing, nuclear export, stability, translation or decay.

N^6^-methyl-adenosine (m^6^A) modification of mRNA is prevalent across organisms and represents a mechanism for post-transcriptional gene regulation. Emerging evidence suggests m^6^A modification intervenes mRNA alternative splicing, translational efficiency and stability [28–30]. Being the m^6^A ‘writer’, methyltransferase-like 3/14 (Mettl3/14) regulate the expression and splicing of spermatogenesis-related genes required for alternative splicing of genes for SSCs maintenance (*Dazl*) and differentiation (*Sohlh1*), and its deficiency compromises spermatogonial differentiation [31–33]. The SSCs vanishing and spermiogenesis defects induced by *Mettl3/14* depletion is reported by another study [32]. A glimpse of m6A in RNA biology is depicted in Fig. 4.

Alternative splicing confers gene pleiotropy of several determinants in SSCs. The stem cell factor (SCF) c-kit is specifically expressed in differentiating spermatogonia to support survival and proliferation of pre-meiotic germ cells [34]. However, the truncated form of c-kit (TR-kit) is enriched in post-meiotic spermatozoa with an unexplored relevance [35]. p73, a member of p53 family of transcription factors, has two major isoforms, TAp73 (with transcription transactivation (TA) domain) and ΔNp73 (without TA domain) [36]. Deficient in *TAp73* increases DNA damage induced spermatogonia death which can’t be phenocopied by ΔNp73 depletion, implying functional non-redundancy between them [37,38].

Several multifaceted RNA helicases, such as MOV10 and DDX5, have been implicated in alternative splicing in spermatogonia [39,40]. MOV10 is highly enriched in spermatogonia and involves in alternative splicing and miRNAs processing to regulate spermatogonia self-renewal. MOV10-mediated splicing initiates in nucleus, where it cooperates with prototypical splicing factors, such as SRSF1 and DDX5, to control RNA fates [39]. In contrast to MOV10, MOV10-like-1 (MOV10l1), a putative RNA helicase, is primarily expressed in pachytene spermatocyte and its deficiency causes meiotic arrest, transposons activation, and depletion of iPiwi proteins MILI and MIWI-associated perinatal piRNAs [41].

## Alternative splicing in spermatocyte meiosis

The differentiated type B spermatogonia further differentiates into prepachytene spermatocytes which pass through the blood-testis barrier (BTB) becoming residents at the apical compartment wherein they transform into primary spermatocytes for meiosis [42]. Multiple proteins in splicing machinery are more expressed in pachytene spermatocytes, and alternative splicing network is intensively reprogrammed, including downregulation of PTBP1 and hnRNPA1, and upregulation of Sam68, Nptb, Tra2β and T-STAR during mitosis-meiosis transition [24,43], indicating alternative splicing is critical for germline commitment.

### Alternative splicing in mitosis-to-meiosis transition

Pachytene spermatocytes undergo two consecutive meiotic divisions without an interval of DNA synthesis to produce haploid gametes [43]. BCAS2 participates in mitosis-to-meiosis transition during spermatogenesis via harnessing the splicing of spermatogenesis-related genes, such as *Dazl, Ehmt2 and Hmga1*, and its deficiency causes alternative splicing catastrophe in male germline leading to compromised spermatogenesis with rare meiotic spermatocytes [26]. The RNA-binding protein (RBP) DAZL serves as an intrinsic ‘meiosis’ promoting factor by targeting mRNAs for germ cell development including *Mvh, Sycp3, Tpx-1* and *Tex19.1,* and thereby regulates their translation [3232^44^32^45^, ^46^]. *Bcas2* depletion favours the exon 8 deleted DAZL (Dazl-Δ8) production instead, which dwindles the functional full-length DAZL reservoir [26]. Spo11, a Topoisomerase II (TOP2)-like transesterase generates DNA double-strand breaks (DSBs) and initiates homologous recombination during meiotic prophase [47], encodes two isoforms that differ fin exon 2 skipping (Spo11α) or inclusion (Spo11β) through alternative splicing. Spo11β is highly expressed in mid-preleptotene and leptotene spermatocytes, and responsible for most DSBs formation. Whereas Spo11α is expressed in mid-pachytene spermatocytes, and likely be essential for efficient XY pairing at later stage [47,48]. The concerted action of RNAPII and specific splicing factors may underlie the spatiotemporal regulation of splicing pattern whereby RNAPII and hnRNPH determines Spo11 isoform expression [49].

Importantly, RBPs, by interacting with distinct partners, broadens the regulatory kingdom of alternative splicing. Ran-Binding Protein 9 (RanBP9), also called RanBPM, a multi-modular scaffolding protein belongs to RBP family, is essential for mammal gametogenesis as male and female lacking *RanBP9* are both sterile [50,51]. Defects in spermatocytes and spermatids due to persistent DNA damage and aberrant expression of key spermiogenic genes are features of *RanBP9* deficient mice [50]. RanBP9 together with other splicing factors (e.g. SF3B3 and HNRNPM) and nuclei/cytoplasm shuttling poly (A) binding proteins (e.g. PABPC1 and PABPC1) regulate spermatogenic pre-mRNA processing in spermatocyte and mRNA metabolism in spermatid, respectively [50,52]. More interestingly, by interacting with RanBP9 and SF3B, the core pacemaker CLOCK is assembled into alternative splicing machinery during murine spermatogenesis [53], providing a new insight of circadian rhythm in mammallian reproduction.

The Src-associated substrate in mitosis of 68 kDa (Sam68), a signal transducer and RBPs activator, is a ubiquitous splicing factor for specific mRNAs. *Sam68* ablation compromises pachytene spermatocytes survival and spermatocytes meiosis [54]. In addition, Sam68 facilitates recruitment of polysome for mRNAs translation, which is exemplified by NEDD1, a centrosome protein; SPDYA, a cell cycle regulator; and SPAG16, a cytoskeletal protein for sperm motility [54]. In addition, Sam68 conjugates with phosphorylated RNA polymerase II (RNAPII) at transcriptionally active chromatins, by which it modulates the splicing of *Sgce* exon 8 in meiotic spermatocytes via affecting phosphorylated Pol II and U2AF65 recruitment, leading to exon skipping [55].

Beyond the ubiquitously expressed splicing factors, a plethora of testis-specific ones, such as RNA-binding motif on Y chromosome (RBMY), heterogeneous nuclear ribonucleoprotein G-T (hnRNPG-T), signal transduction and activation of RNA (T-STAR) and homeodomain-interacting protein kinase 3 (HIPK3) have been identified in mammalian testis [56,57]. RBMY and hnRNPG-T, by interacting with T-STAR and Sam68, promotes exonic splicing of testis-specific *TLE4-T* in cooperation with SR-like protein TRA2b [56,58]. Furthermore, RBMY antagonizes the activity of TRA2b and SR protein 9G8 in the case of excluding a testis enriched exon from Acinus gene [59]. Although not testis-specific, the RNA recognition motif (RRM)-containing protein 11 (RBM11), a brain and cerebellum enriched splicing factor, shows high abundance in meiotic spermatocytes and post-meiotic spermatids that deserves further functional investigations [60].

### Alternative 3ʹUTR selection in spermatocyte meiosis

In parallel to exon skipping by which alternative splicing generates transcript variants, the choice between mutually exclusive exons, use of alternative splice sites and intron retention intricately create transcripts differ in untranslated region (UTR). The length of 3ʹUTR influences mRNAs stability and translation likely due to longer 3ʹUTRs have more propensity of RBPs binding while shorter ones loss the priority [61,62], making 3ʹUTR length a mRNAs fate definer. Interestingly, testis has more enrichment for transcripts with shorter 3ʹUTR [63]. Spermatogonia and meiotic spermatocytes mainly transcribe transcripts with longer 3ʹUTR. In contrast, transcripts with shorter 3ʹUTR increasingly enrich from late pachytene spermatocytes to spherical spermatids, and migrate into ribonuclear protein particles (RPPs) for stabilization and translational suppression [64].

Intron retention (IR) is the most represented pattern of alternative splicing that contributes to the plasticity of transcriptome and the regulation of gene expression [65,66]. IR is also the most enriched form of AS during spermatogenesis. High-throughput transcriptome profiling revealed a robust IR phenomenon in meiotic spermatocytes, intron-retaining genes are weak in splice sites and are enriched in functional categories strongly relevant for gamete function, and the intron-retaining transcripts (IRTs) were exclusively localized in the nucleus [67]. In general scenarios, IR causes transcript instability in mammals, leading to the quenching of gene expression during cell differentiation or in response to cellular stress [68–71]. While a ‘positive’ role for IR is described during spermatogenesis. IR could stables and retains RNAs in the nucleus of meiotic cells for days after their synthesis prior to being recruited onto polysomes for translation. Interestingly, those retained introns could experience splicing in post-meiotic cells [67]. The observations captures the pivotal role of IR in conferring the spatio-temporally differential expression of specific genes during spermatogenesis. One key feature of mammalian spermatogenesis is the discontinuous nature of transcription. In particular, de novo synthesis of mRNAs is ceased in post-meiotic cells due to exchange of histones with protamines and tight compaction of the chromatin [72]. Hence, IR might constitute a machinery uncoupling transcription and translation of abundant mRNAs that are required for proper sperm function.

Testis-specific double bromodomain-containing protein (BRDT), a bromodomain and extra-terminal motif (BET) family member, is the only BET protein highly expressed throughout the transcriptionally active pachytene stage and in haploid spherical spermatids along with BRD2/3 which is required for production of fertile sperms [73]. BRDT has multifunctions during spermatogenesis including transcriptional regulation in pachytene spermatocytes and spherical spermatids, and generation of 3ʹUTR truncated transcripts in post-meiotic spermatids [74]. Over 400 genes are differentially expressed in spherical spermatids carrying *Brdt* bromodomain deletion (*Brdt^ΔBD^*) with the up-regulated genes are enriched for splicing genes [74].

One commonality to most of those splicing genes including Serine/arginine-rich splicing factor 2 (*Srsf2*), Asp–Glu–Ala–Asp (DEAD)-box polypeptide 5 (*Ddx5*) and heterogeneous nuclear ribonucleoprotein K (*Hnrnpk*) is the 3ʹUTR truncation while the major spliceosome components, such as TAR DNA binding protein (*Tardbp*), are with longer 3ʹUTR accompanied by reduced protein expression 74. That is, the translation is conductive to those with shorter 3ʹUTR, which represents another post-transcriptional regulatory mechanism of spermatogenesis. Furthermore, *Brdt* depletion severely disrupts chromatin modifications involved in meiotic sex chromosome inactivation (MSCI) which epigenetically reprograms and transcriptionally ceases XY body, the chromatin configuration, and the crossover dynamics in spermatocytes, entitling BRDT an essential reprogramming and chromatin organization factor in spermatocytes meiosis [75]. However, whether BRDT directly or indirectly maintain MSCI needs further investigations.

### Splicing intertwines with mRNA modification in spermatocyte meiosis

Though with limited abundance, emerging evidence suggests mRNA m^6^A modification, which is written by methyltransferase and erased by demethylase, is of biological importance, such as intertwine with 3ʹUTR splicing in spermatogenesis [76] (Fig. 4). ALKBH5, a m^6^A demethylase, mediates m^6^A erasure in the nuclei of spermatocytes and spherical spermatids, and favours the production of transcripts with longer 3ʹUTR. *Alkbh5* deletion causes hyper-m^6^A in mRNA and aberrant splicing to produce transcripts with shorter 3ʹUTR, both leading to instability of mRNA [77]. *Alkbh5* deficient mice are sterile due to compromised spermatocytes meiosis [76]. The phenotype somehow recapitulates that of in *Mettl3* deficient mice. Mettl3 is a m^6^A writer, and its deletion causes more severe meiotic arrest of spermatocytes at zygotene/zygotene-like stage [33]. The evidence posits that the homoeostasis of m^6^A modification is vital for germline commitment.

Attractively, novel m^6^A modification factors in spermatocytogenesis are emerging. YT521-B homology domain-containing 2 (YTHDC2), a m^6^A ‘reader’ that recognizes m^6^A marks, functions in RNA splicing, mRNA decay and translation control, and is essential for male fertility [30,78,79]. YTHDC2 in mouse germ cell interacts with Meiosis specific with Coiled-coil domain (MEIOC) an important meiosis-specific protein [80]. Beyond being the m^6^A ‘reader’, YTHDC2 has 3ʹ-5ʹ RNA helicase activity to regulate the abundance of m^6^A-modified transcripts, which ensures the programming of mitotic spermatogonia to meiotic spermatocytes [79,81].

### Alternative splicing in spermiogenesis

The final differentiation of spermatogonia is an extraordinarily integrated process termed spermiogenesis, through which the haploid spherical spermatid (rST) transforms into highly specialized tadpole-shaped spermatid prior to leaving seminiferous epithelium for further maturation in epididymis.

Spermatids elongation is one hallmark for spermatogenesis. Transcription cessation gradually takes place from rST onwards, but alternative splicing again deems its power. PTBP2 is a key splicing factor in nervous system, but also functions in germ cell. PTBP2 persists through spermatocytes to elongating spermatids, and spermatogenesis of *Ptbp2* depleted germ cells arrests at spermatid differentiation accompanied by apoptotic death of spermatocytes [82,83]. Interestingly, disrupted cytoskeleton architecture is seen in Sertoli cells [82], suggesting a communication between these two cell types. Another hallmark for spermiogenesis is the extensively nuclear remodelling, during which histone-transition nuclear proteins (TNPs)-protamine exchanging occurs, leading to chromatin hypercompaction required for spermatids fully maturation [84,85]. Prior to removal, histones remain hyperacetylated with which BRDT associated. BRDT anchors to double-acetylated H4 (K5ac and K8ac) vis its first bromodomain (BD1), and directs genome-wide histone replacement [86,87]. Alternatively, BRDT interacts with Smarce1 (BAF57) a member of SWI/SNF family of ATP-dependent chromatin remodel complexes to fuel chromatin remodelling [88]. Its importance in meiosis and post-meiotic nuclear remodelling make BRDT a promising male contraception target or infertility therapy.

Interregulation of histone modification and alternative splicing is evident by chromatin-binding protein dependent recruitment of splicing factors [89]. MORF-related gene on chromosome 15 (MRG15) is a multifunctional chromatin organizer that binds to lysine 36 methylated H3 (H3K36) of transcriptionally active genes to facilitates alternative splicing [89]. In spherical spermatids MRG15 co-localizes with splicing factors PTBP1/2 at H3K36me3 sites between exons, and spermatogenesis in *Mrg15* null mice arrests at spherical spermatid stage but with normal histone methylation [90], which functionally links histone methylation with alternative splicing for spermatids elongation. However, the splicing defects in *Ptbp2* and *Mrg15* null spermatids are non-overlapping, suggesting MRG15 and PTBP2 are specialized for splicing events.

RBM5 (RNA binding motif 5) is another novel splicing factor highly enriched in testis, especially in spermatocytes and spherical spermatids, and is required for spermatid differentiation. RBM5 directly complexes with several partners implicated in pre-mRNA splicing, such as hnRNP, SR proteins, SFPQ and RNA helicase DDX5, and several RBPs include PABP1, DDX4, PSPC1 and ELAV1 that regulates various RNA processing events are also RBM5 putative partners [91]. In *Rbm5* mutant mouse, splicing defects alter the expression of genes involved in MAPK/ERK and JAK/STAT signalling required for spermatid differentiation including germ cell adhesion, spermatid head shaping, and acrosome and tail formation [90]. More beyond, apoptosis-related genes, such as *FAS, Caspase2* and *c-FLIP* are additional splicing clients of RBM5 [92,93].

The formation of acrosome, a Golgi-derived exocytotic organelle the contents which are indispensable for natural fertilization, is another remarkable event in spermiogenesis. The acrosomal protein ACRBP/sp32 is a binding protein for precursor ACR (proACR) and intermediate ACR [94,95]. In mammals, alternative splicing generates two variants of ACRBP-W and ACRBP-V5, respectively [95,96]. *Acrbp* null mice lacking both isoforms loss fecundity due to acrosome fragmentation. ACRBP-V5 functions in formation and configuration of acrosomal granules during early spermiogenesis while ACRBP-W functions in retention of the inactive proACR in acrosome until acrosomal exocytosis [96], further exemplify how alternative splicing diversifies gene function by producing transcript variants.

## miRNA in regulation of testicle functionality

Fertility is constantly challenged by environmental insults, such as malnutrition, diabetes, stress, infections and xenobiotics. Numerous miRNAs have either ubiquitous or developmental dependent expression in testis, and regulates a myriad of events by targeting genes in different pathways (Table 1). We highlight two conserved stress responsive pathways with miRNA association.

### miRNA and Sirtuin signalling

Sirtuins (Sirt1-7), a family of NAD^+^ dependent deacetylase, have been in limelight over recent years due to their activities towards diverse pathways coping with cellular energetic, metabolic and redox crises [97]. Among them, Sirt1 a nuclear protein has gained most attentions, and potent activators and inhibitors against Sirt1 are commercially available. Pharmacological and genetic interventions modulating Sirt1 activity has deemed its cytoprotective roles by targeting over 50 proteins, such as HDAC1/ATM/XPA (DNA repair), LKB1/AMPK (autophagy), p53 (apoptosis), Nrf2 (antioxidative) and PGC1α (mitochondria biogenesis) [97,98].

Converging evidence suggests Sirt1 is a determinant for male and female fertility. Sirt1 is highly expressed from spermatotonia to spherical spermatids [99]. *Sirt1^−/−^* mice are sterile with decreased testes size and rare presence of spermatozoa due to spermatogenic arrest at late-meiotic prophase followed by germ cells degeneration, and even a scarcity of spermatozoa can be retrieved they are immotile with morphological anomalies [98,100]. P53 activity is attenuated by Sirt1 mediated deacetylation [97], germ cells apoptosis seen in *Sirt1^−/−^* mice, therefore is ascribe to up-regulated p53 activity. Upon oxidative stress, Sirt1 deacetylates and activates PGC1α to augment antioxidative response [97]. In this regard, oxidative stress is an alternative causative of p53 activation in *Sirt1^−/−^* mice.

Notably, spermatogenesis defects in *Sirt1^−/−^* mice could be a consequence of dysfunctions in hypothalamic-pituitary axis (HPA) [98]. HPA releases gonadotropins, follicle-stimulating hormone (FSH) and luteinizing hormone (LH) to stimulate the two independent but intertwined testicular events, spermatogenesis and androgenesis, respectively. Sirt1 deacetylates cortactin to drive *in vitro* GnRH neuronal migration, and GnRH neurons in *Sirt1^−/−^* mice loss migration from vomeronasal organ to colonize in hypothalamus, leading to hypogonadotropic hypogonadism reminiscent of human congenital Kallmann’s Syndrome that causes male infertility [1,101]. The direct relevance of Sirt1 in spermatogenesis is confirmed by germ cell specific *Sirt1* knockout (*Sirt1^cKO^*) mice whereby novel roles of Sirt1 in acrosome biogenesis and histone to protamine transition are discovered [100].

Multiple miRNAs that target Sirt1 mRNA are reported. Given its relevance to reproductive health, perturbation in Sirt1 activity might underlie the molecular bases for fertility impairments, which is evident by miR-34a in diabetes mellitus (DM) induced testicular apoptotic cell death (TACD) [102]. Obesity and diabetes mellitus are prevalent epidemics that are detrimental for reproductive health. MiR-34a expression is up-regulated in Streptozotocin (STZ)-DM model, and is associated with TACD by targeting Sirt1 mRNA. Inactivation of miR-34a or/and activation of Sirt1 ameliorates cellular stresses and TACD seen in STZ-DM mice [102]. Environmental pollution is another health concern as numerous contaminants including Di-(2-ethylhexyl) phthalate (DEHP) and Bisphenol A (BPA) have more or less developmental and reproductive toxicity largely by triggering redox catastrophe. DHEP exposure increases miR-181a expression while decreasing Sirt1 expression in rat testis [103]. While the correlation is proposed, whether Sirt1 is targeted by miR-181a and, if so, their relevance to DHEP induced testicular toxicity remains unknown.

Besides Sirt1, Sirt2-7 are also of cellular importance. Sirt2 is primarily in cytoplasmic where it deacetylates ɑ-tubulin and checkpoint protein BubR1 to regulate cell division while Sirt3-5 are mitochondrial proteins to maintain mitochondrial functionality [104–106]. Sirt6 is a nuclear protein for DNA repair, and its deficiency causes genomic instability and precocious ageing [107,108]. Sirt7 is also a nuclear factor that shapes nuclear-encoded mitochondrial genes [109]. Elucidating the role of Sirtuins and their epigenetic regulations maybe helpful for male fertility improvements.

### miRNA and Nrf2 signalling

Nuclear factor erythroid 2-related factor 2 (Nrf2) is a ubiquitous stress-rectifying transcription factor that harnesses a spectrum of signalling involved in regulation of redox homoeostasis, autophagy, detoxification, mitochondrial biogenesis and metabolic reprogramming [110–113]. Nrf2 has seven functional Nrf2-ECH homology domains (Neh1-7). Neh1 heterodimerizes with Maf proteins for efficient binding to target genes. Neh2 interacts with DLG/ETGE domain of Kelch like-ECH-associated protein 1 (Keap1). Neh3-5 promotes its transactivation. Neh6 regulates its stability while Neh7 mediates its repression by retinoic X receptor alpha (RXRα). In basal settings, Nrf2 activity is suppressed by canonical Keap1/Nrf2 pathway and noncanonical GSK3β/Nrf2 pathway. Keap1 is a redox sensitive adaptor for Keap1/Cul3/Rbx1 ubiquitin ligase E3 complex that presents Nrf2 to proteasomal degradation while phosphorylation by GSK3β predisposes Nrf2 to be recognized by ubiquitin ligase E3 βTrCP for its degradation [112]. Upon oxidative stress, Keap1 oxidation (Cys151/273/288), and GSK3β inhibitory phosphorylation by PI3K-Akt liberate Nrf2 from the inhibitory complex and thereby augments its activity [110].

Generally, Nrf2 results in favourable outcomes, however, its hyperactivation is pronounced by chemotherapeutic drug resistance [114]. Nrf2 activity is regulated at multiple levels. At transcriptional level, promoter modifications and several transcription factors (e.g. NF-kB and Myc) regulate Nrf2 transcription [110]. miRNA constitute the major post-transcriptional regulator of Nrf2. A plethora of miRNAs that target Nrf2 (e.g. miR-144) decrease its expression [115], whereas miRNAs that target Keap1 (e.g. 200a) and Cul3 (e.g. miR-101) potentiate its activity [116,117]. Beyond miRNA, alternative splicing and RBP (e.g. HuR) also regulate Nrf2 activity. A spliced Nrf2 transcript lacking Keap1 binding domain causes Nrf2 stabilization and constitutive activation [118]. Post-translational regulation of Nrf2 is diversified by protein modifications, such as sumoylation, phosphorylation and acetylation [110]. For example, phosphorylation by kinases like PERK, JNK and PKCδ activates Nrf2 through deconjugating it from Keap1, while phosphorylation by Fyn inactivates it through promoting its nuclear export [112]. In addition, chaperons including p62 that disrupt Keap1-Nrf2 interaction also augment Nrf2 activity. p62, a selective autophagy adaptor with Keap1 binding property, sequesters Keap1 for autophagosomal degradation and thereby activates Nrf2 [119]. Phase condensation of Keap1 by p62 might be a novel mechanism of Nrf2 activation [120]. Intriguingly, p62 is a target of Nrf2, representing a positive feedback augmenting Nrf2 activity [119].

Although well studied in other fields, Nrf2 signalling in reproductive biology is mysterious. Even with decreased foetal weight and increased susceptibility to hyperoxia, *Nrf2^−/−^* mice are viable presumably due to the compensation effect of Nrf1 as mice carrying combinatorial deletion for *Nrf1* and *Nrf2* die days after birth [121]. Interestingly, *Keap1^−/−^* mice with Nrf2 hyperactivation also die before weaning [122]. *Nrf2^−/−^* mice have compromised fecundity with declined spermatid output due to oxidative damage [123]. In addition, Nrf2 is abundant in human spermatogonia, spermatocytes and spermatids. Lower Nrf2 expression is associated with weakened sperm motility, and single nucleotide polymorphisms in Nrf2 promoter, which reduces its expression are observed in patients with oligoasthenozoospermia [124,125]. MiR-101-3p that targets Nrf2 further emphasizes its testicular protective role. Torsion of testicular vascular pedicle is a common urologic injury that causes testicular damage or even orchiectomy. Timely detorsion intervention restores testicular blood circulation, but ischaemia–reperfusion (I/R) injury is an inevitable phenomenon. MiR-101-3p is up-regulated in I/R injured rat testes, and inhibition of miR-101-3p and up-regulation of Nrf2 ameliorate the oxidative damage to *in vitro* hypoxic/reoxygenation modelized Leydig cell (TM3) [116]. Further scrutiny of miRNA-Nrf2 network is needed to better understand Nrf2 importance in reproductive fitness.

## miRNAs in multigenerational repercussions of paternal experiences

**Figure 5.**
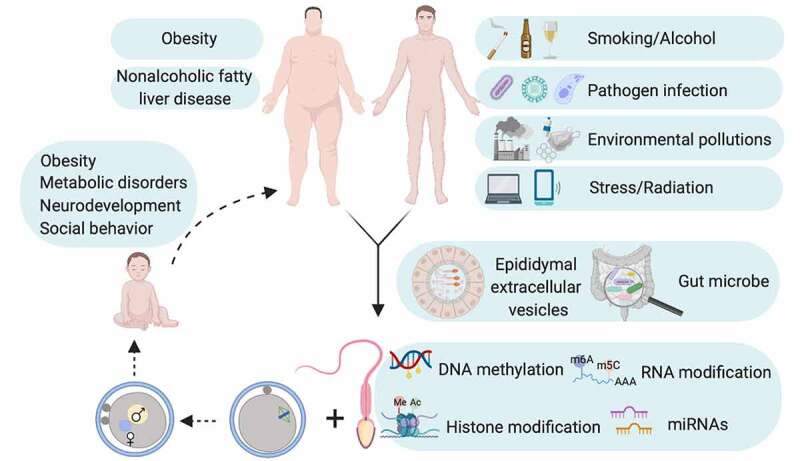
Intergenerational cycle of disease. Unfavourable paternal health may reprogram epigenome in gamete probably through epididymal extracellular vesicles and/or gut microbe and thereby creates intergenerational repercussions of paternal pathologies. Diagram is conceptualized based on studies on mouse model.

The study of paternal experiences and heritability to offspring is a topic of interest since long. While previously considered has limited contribution except paternal genome to embryo in light of the facts that (1) negligible size compared with oocyte; (2) transcriptionally inert; (3) limited remnant of cytoplasm with compromised translation machinery, sperm now is believed to preserve important epigenetic codes to shape embryonic development and offspring health. Indeed, embryos of *Dicer* cKO sperm origin have preimplantation defects that can be ameliorated by injection of naive sperm RNAs [16]. More remarkably, injection of small RNAs, more specifically miRNA sized small RNAs, from cauda epididymis derived epididymosome completely rescues pleiotropic defects in caput epididymal sperm derived embryos [15]. In addition, paternal pathologies can reprogram sperm epigenome to influence offspring phenotype as healthy zygote injection of up-regulated sperm miRNAs of stressed male recapitulates pathologies in offspring of stressed male [20,21]. The influence on sperm epigenome of environmental pertubations that initiates intergenerational cycle of disease susceptibility Is captured in Fig. 5.

Given the prevalence of obesity pandemic, the influences on offspring health of parental obesity have attracted considerable attentions. Obesity up-regulates miR-122/155 in men’s sperm [126], and human epidemiological studies reveal that overnutrition in paternal grandfathers increases mortality risk in grandsons [127]. In high-fat diet (HFD) mouse model, paternally inherited pathologies-insulin resistance, glucose intolerance, obesity and fertility impairments-are observed in F1 and F2 offspring. HFD feeding up-regulates 11 miRNAs and down-regulates 2 miRNAs in paternal sperm, but those alternations are not evident in sperm of F2 offspring, albeit with metabolic and fertility phenotypes [128]. In another obesity mouse model, paternal obesity initiates inheritance of metabolic pathologies across three generations with 15 up-regulated and 9 down-regulated miRNAs are identified in F1 offspring’s sperm [129]. In HFD rat model, HFD exposure reprograms epigenome in paternal sperm and likewise affects offspring’s metabolic health. Among the 15 differentially expressed miRNAs, miR-let-7 c-5p a regulator of metabolism signalling is of particular interest as its expression is also altered in offspring metabolic tissues [130]. Recent injection studies manifest the bona fide importance of miRNA in paternally obesity induced multigenerational repercussions. Obesogenic western diet feeding up-regulates 11 miRNAs, including miR-19b, and down-regulates 2 miRNAs in sperm, and naive zygote injection of obese sire’s sperm RNAs or miR-19b alone initiates metabolic pathologies seen in obese male’s progeny [131].

Of note, physical intervention is a common recommendation for preconception care, but paternal long-term physical activity predisposes offspring to higher susceptibility to HFD induced pathologies of glucose intolerance and insulin resistance, and modifies DNA methylation and miRNA repertoire in paternal sperm [132]. That is, a harmonized paternal lifestyle matters for offspring health. Like obesity, paternal exposure to famine and undernutrition also adversely affects offspring health. In human, famine experience in paternal grandfathers increases obesity susceptibility in grandsons [19]. In mouse, low-protein diet (LPD) feeding reprograms sncRNA repertoire, such as down-regulation of several miR-let-7 species including let-7 c, in paternal sperm, and impairs offspring’s liver cholesterol biosynthesis with which associated genes are dysregulated [133].

Traumatic stress is an environmental stimuli living with most individuals that has intergenerational influences. Early life trauma correlates with down-regulation of several miR-34/449 family members that program neurodevelopment and spermatogenesis, in sperm of both humans and mice, and the phenomena persist over generations in mice [134]. When exposing adult male mice to chronic stress, 9 miRNAs are up-regulated in sperm, and injection of a mixture of these miRNAs but not individual miRNA into naive zygotes recapitulates psychiatric disorders of offspring of stressed male [20]. Post-spermatogenic sperm is transcriptionally quiescent, but during epididymal maturation its sncRNA signature undergoes reprogramming as sperm receives signals from epididymis derived epididymosomes [4]. Caput epididymal and cauda epididymal sperm has distinct miRNA payload. Injection of small RNAs or small RNAs of miRNA size from caudal epididymosomes into caput epididymal sperm derived zygotes astonishingly rescues their preimplantation defects and postimplantation lethality [15]. Importantly, epididymosomal contents are vulnerable to environmental stimuli as corticosterone a stress hormone dramatically alters proteome and transcriptome in epididymosomes from epididymal epithelial cells. Intracytoplasmic injection of sperm pre-incubated with those epididymosomes produces offspring with impaired neurodevelopment [22]. These findings support Charles Darwin’s pangenesis hypothesis of transferring environmental information from somatic to germline.

Over evolution, pathogens are co-evolving with us. They hijack host cellular machinery and reshapes host epigenome to create a conducive microenvironment for its pathogenesis [135]. Numerous pathogens are detrimental to brain and reproductive function [21,136]. It is known that maternal toxoplasma infection affects offspring mental health [137], but the effect may pass through maternal–foetal interaction and/or maternal grooming behaviour other than germline. The intergenerational influences of toxoplasma infection through germline has been reconciled very recently. Paternal toxoplasma infection increases sperm abnormalities and deregulates 174 miRNAs in sperm. Naive zygote injection of sperm small RNA from infected mice recapitulates psychiatric disorders of offspring of infected male [21]. The finding will refresh our conception plan when carrying pathogenic infection if the similar phenomenon occurs in human.

## Conclusion remarks and perspectives

Infertility affects nearly 48.5 million reproductive-age couples worldwide, and around 2% of men present with compromised sperm parameters [1]. Spermatogenesis, an extremely elaborate process taking ~74 days in human, requires concerted regulation of the germ cell-specific transcriptome of exceptional diversity. The origin of male infertility, such as oligozoospermia and necrozoospermia, in most cases, remains unexplained [1], which fascinates the missing non-genetic hereditary codes. Alternative splicing that produces functionally distinct mRNA variants, and miRNA that silences mRNA translation regulate events throughout spermatogenesis, and their dysfunction implicates in non-genetic aetiology of male infertility. Interestingly, they can inter-regulate each other [138], which further complicates RNA regulation network.

Oxidative stress represents a culprit of ageing and many pathologies including infertility [98,112]. Although assisted reproductive technology, such as intracytoplasmic sperm injection, to some extents overcomes male infertility, the injected sperm may carry stress-induced damage. As discussed, Sirt1 and Nrf2 pathways are conserved pro‐survival mechanisms. Sirt1 activating compounds, such as resveratrol and NAD^+^, have promising health benefits and can juvenilize metabolism and rhythm system [139,140]. Nrf2 is a tier regulator of oxidative stress, and natural products that activates Nrf2 have positive effects on disease treatment [141]. Whether chronic activation of these pathways before or during conception improves pregnancy outcome especially in people with health problem and if so, the long-term safety of those compounds are interesting questions. More understanding of these pathways and their epigenetic regulations are definitely helpful for development of clinical interventions.

In testes, Sertoli cells in seminiferous tubule support germ cells development, and construct an immuno-privilege niche for them [142]. Sertoli cells are sensitive to blood signals but highly resistant to apoptosis. That is, Sertoli cells are granted with potent stress responsive mechanisms. Are the aforementioned pathways are relevant to Sertoli cells functionality remain elusive. LC3-dependent phagocytosis of millions of apoptotic germ cells on a daily base by Sertoli cells is key to spermatogenesis [142]. Autophagy components, such as p62 and LC3, are targets of Nrf2 [112,119], and Nrf2 promotes LC3-dependent phagocytic activity of retinal pigment epithelium [141]. Thus, Sertoli cells may hijack similar mechanism for phagocytosis, which warrants further investigations.

Although miRNA matters for epigenetic inheritance, it is not the sole reason (Fig. 5). Mammalian sperm is unique in tRNA-derived fragment RNAs (tRFs), and paternal obesity reprograms tRFs signature in sperm of offspring [13,143,144]. In addition, HFD feeding increases the expression of Dnmt2 a tRNA methyltransferase in paternal sperm. Dnmt2 deletion abolishes transmission of paternal HDF-pathologies [145], suggesting tRNA is another important vector of intergenerational inheritance, which needs further investigations in different animal models. More intriguingly, gut microbe, the rising star in biology, can affect reproductive health and host m^6^A mRNA modification [146–148], implicating gut microbe may regulate sperm epigenome, which will be a tantalizing area. While studies on epigenetic inheritance are flourishing, one principle question is how phenotypes are transmitted across generations. RNA mediated DNA methylation may give an answer [4,149]. Thus, cracking the epigenetic network at multiple layers will advance not only the understanding of how our unfavourable lifestyle impinges on offspring health through germline but also the development of diagnostic and therapeutic algorithms for preconception care and prevention of intergenerational transmission of diseases.
